# The psychotropic effect of vitamin D supplementation on schizophrenia symptoms

**DOI:** 10.1186/s12888-021-03308-w

**Published:** 2021-06-15

**Authors:** Aras Neriman, Yilmaz Hakan, Ucuncu Ozge

**Affiliations:** 1 Department of Psychiatry, Samsun Mental Health and Disorders Hospital, Samsun, Turkey; 2 Department of Psychiatry, Trabzon Kanuni Training and Research Hospital, Trabzon, Turkey; 3 Department of Endocrinology and Metabolism, Karadeniz Technical University, Faculty of Medicine, Trabzon, Turkey

**Keywords:** Schizophrenia, 25OHD, Vitamin D deficiency, Vitamin D insufficiency, Positive and negative symptoms, Cognitive symptoms

## Abstract

**Background:**

Schizophrenia is a multifactorial disease involving interactions between genetic and environmental factors. Vitamin D has recently been linked to many metabolic diseases and schizophrenia. Vitamin D plays essential roles in the brain in the context of neuroplasticity, neurotransmitter biosynthesis, neuroprotection, and neurotransmission. Vitamin D receptors are demonstrated in most brain regions that are related to schizophrenia. However, very few studies in the literature examine the effects of 25-hydroxyvitamin D (25OHD) on schizophrenia symptoms.

**Methods:**

This study aimed to examine the effects of vitamin D replacement on positive, negative, and cognitive symptoms of schizophrenia. Serum 25OHD levels of 52 schizophrenia patients were measured. SANS and SAPS were used to evaluate the severity of schizophrenia symptoms, and the Wisconsin Card Sorting Test: CV4 was used for cognitive assessment. The study was completed with 40 patients for various reasons. The patients whose serum 25OHD reached optimal levels after vitamin D replacement were reevaluated with the same scales in terms of symptom severity. The SPSS 25 package program was used for statistical analysis. The Independent-Samples t-test was used to examine the relationship between the variables that may affect vitamin D levels and the vitamin D level and to examine whether vitamin D levels had an initial effect on the scale scores.

**Results:**

The mean plasma 25OHD levels of the patients was 17.87 ± 5.54. A statistically significant relationship was found only between the duration of sunlight exposure and 25 OHD level (*p* < 0.05). The mean SANS and SAPS scores of the participants after 25OHD replacement (23.60 ± 15.51 and 7.78 ± 8.84, respectively) were statistically significantly lower than mean SANS and SAPS scores before replacement (51.45 ± 17.96 and 18.58 ± 15.59, respectively) (*p* < 0.001 for all). Only the total attention score was significantly improved after replacement (*p* < 0.05).

**Conclusion:**

The data obtained from our study suggest that eliminating the 25OHD deficiency together with antipsychotic treatment can improve the total attention span and positive and negative symptoms in schizophrenia. The 25OHD levels should be regularly measured, replacement should be started when necessary, and the patients should be encouraged to get sunlight exposure to keep optimal 25OHD levels.

## Introduction

Schizophrenia is a multifactorial disease involving interactions between genetic and environmental factors. Although various theories have been proposed to explain its etiology, the most accepted one is the neurodevelopmental model. According to this model, based on a genetic liability, environmental factors such as adverse events or potentially harmful stressors in the perinatal period disrupt the brain’s normal maturation process and neuronal development. After a certain latent period, the clinical symptoms appear in adolescence or young adulthood [1]. Schizophrenia prevalence varies in geographic regions. Environmental risk factors such as a high schizophrenia prevalence at higher latitudes, in winter/spring births, and darker-skinned people support the role of vitamin D in the etiology of schizophrenia [2–5].

Almost all (90–95%) of vitamin D, the only vitamin that can be synthesized in the human body, is synthesized in the skin by the effect of direct sunlight. The amount taken with diet is minimal. Provitamin D turns into vitamin D_3_ in the skin with the effect of ultraviolet-B rays. Vitamin D_3_ undergoes hydroxylation first in the liver to generate 25-hydroxyvitamin D (25OHD) and then in the kidneys to generate the physiologically active form, 1,25-dihydroxyvitamin D (1,25 OH-2D). It binds to the vitamin D receptor (VDR) in tissues [3, 6–8]. The amount of synthesized vitamin D depends on age, skin color, season, ethnicity, and duration of sun exposure, the latitude of the place of residence, use of sunscreen, clothing style, and the size of the skin surface exposed to the sun [8, 9]. The best parameter to show vitamin D level is the serum 25OHD level [10].

While a major role of vitamin D concerns calcium balance and bone metabolism, it also serves several other metabolic functions. Recent studies have shown associations between vitamin D and autoimmune, metabolic, neurological, and allergic diseases and malignancies. Vitamin D also has potential links with psychiatric illnesses such as schizophrenia, autism, attention-deficit/hyperactivity disorder (ADHD), depression, premenstrual syndrome, and anxiety [6, 11–15].

Vitamin D plays essential roles in neuroplasticity, neurotransmitter biosynthesis, neuroprotection, and neurotransmission in developing and adult brains [16–18]. Vitamin D reduces oxidative stress and inflammation caused by free radicals and reactive oxygen metabolites by inducing certain neurotrophic factors [6, 16, 19, 20].

The enzyme that converts vitamin D into the active form (1-α hydroxylase) and VDRs are found in several parts of the body, including the substantia nigra, hippocampus, thalamus, hypothalamus, amygdala, prefrontal gyrus, and cingulate gyrus. This enzyme plays a role in regulating behavior in the central nervous system [11, 14, 19, 21]. In brain imaging studies about vitamin D, inverse relationships were found among vitamin D levels and total intracranial volume, total cortical gray matter, and cerebral white matter volumes [22, 23]. Decreased hippocampal volume in schizophrenia has been shown in several studies. The hippocampus is the brain region with the highest number of VDRs. Vitamin D plays a crucial role in hippocampal cell life through its neuroprotective effect [16].

Exposure to low maternal vitamin D levels in the fetus and low 25OHD levels in the neonatal period were found to increase the risk of developing schizophrenia in later years [17]. Animal studies reported that dopamine metabolism is affected in the brains of rats that had developmental vitamin D deficiency during the gestational period [24]. The risk of schizophrenia was shown to decrease by 77% in people who had taken vitamin D supplements in the first year of life [11].

Vitamin D deficiency was seen in more than half of the adult population and was considered to be at a pandemic level in several publications [25–27]. Vitamin D deficiency is more common in patients with schizophrenia due to factors such as social isolation, lack of movement, smoking, spending less time outside, malnutrition, and disruption of vitamin D synthesis by antipsychotic drugs [2, 7, 19, 28–31].

Studies on vitamin D deficiency in schizophrenia patients usually include the first episode and acute exacerbation periods. Vitamin D levels outside these periods have been rarely studied. Also, there are very few studies in the literature examining the effect of vitamin D replacement on disease symptoms. Therefore, we aimed to investigate the rates of vitamin D deficiency, related factors, and the effect of vitamin D replacement on clinical symptoms and cognitive functions in schizophrenia patients who attended an outpatient treatment facility in our region.

## Material and methods

### Participants

This study was conducted with schizophrenia patients visiting the Community Counseling Service at Trabzon Kanuni Training and Research Hospital. These services are the outpatient mental health clinics serving under the Ministry of Health in Turkey. Patients who are clinically stable with chronic mental diseases (schizophrenia, schizoaffective disorder, and bipolar disorder) are accepted to the center according to official guidelines. Community Counseling Services carry out the medical follow-up and rehabilitation of the patients’. Medication, social needs, and consultancy services are provided to the patients and caregivers. Psychiatrists, psychologists, nurses, and social workers are provide consultancy and home visits are performed by this specially trained team with periodically.

The patients were selected by simple randomization. Written informed consent was obtained from all participants following the explanation of the study procedure. Ethics committee approval for this study was obtained from the local ethics committee.

Structured Clinical Interview for DSM-4 (SCID-I) was administered to patients by a psychiatrist to diagnose schizophrenia. Patients between the ages of 18–65 who were diagnosed with schizophrenia according to the Diagnostic and Statistical Manual of Mental Disorders (DSM-5) criteria were included in the study.

were in an acute exacerbation period, had a history of head trauma, alcohol-substance use disorder, and those using antiepileptics or vitamin D supplements were not included in the study. During the study, patients whose antipsychotic dose or active substance were changed, who did not want to continue the vitamin D replacement therapy, who did not use at recommended doses, or who could not complete the tests were excluded from the study.

### Design and procedure

This study was a case-control study conducted between May 2017 and November 2017 to determine the rates of vitamin D deficiency and related factors in schizophrenia patients and evaluate the effect of serum total vitamin D level on disease symptoms. Blood samples of the patients were taken from May to July to measure serum vitamin D levels. A psychiatrist performed psychiatric evaluation, SCID-I, and clinical scales; a specialist psychologist performed cognitive tests, and an endocrinology and metabolism specialist evaluated the vitamin D levels and replacement therapy.

Fifty-two schizophrenia patients were included in the study; a total of 12 patients were excluded during the course of the study. The reasons for exclusion were as follows: two patients had 25OHD levels within normal limits, two patients did not comply with their current antipsychotic treatment or changed their treatment, three patients did not want to continue the study, three patients did not complete cognitive tests, and two patients did not use vitamin D replacement regularly. Forty patients completed the study.

First, we measured the 25OH-D levels in randomly selected schizophrenia patients. We eliminated schizophrenic patients that are levels of 25OH-D were not low. Clinical assessment scales (SANS, SAPS and WCST-CV) were applied to the patients with low vitamin D levels, before replacement. Vitamin D replacement was performed in appropriate doses, as recommended in treatment guidelines of ‘Vitamin D Deficiency Treatment Guide of Endocrinology and Metabolism Association of Turkey’. Vitamin D replacement was completed in time, because it took time for some patients’ measurements to rise above the threshold level of > 30 mEq/L. Therefore, some patients had to be given additional doses and repeated measurements of vitamin D, may be possible personal differences or absorption problems. Finally, after the vitamin D level reached above the threshold value, we reapplied SANS and SAPS scales and WCST: CV4 again. The significant change seen in clinical symptoms reflects symptomatic improvement after replacement compared to before replacement.

### Instruments

A sociodemographic data form including gender, age, educational status, body-mass index (BMI in kg/m2), smoking, tea and coffee consumption, skin color, sun exposure, daily activity level, clothing style, and diet (frequency of fish consumption) was filled simultaneously at the time of blood sampling. Daily physical activity was classified as time (hour/day) as moderate speed of walking by patients’ self report (Low-(< 1 h/day, moderate-1 h/day, adequate- > 1 h/day).

Ethnic origin was not included in the study because all patients had the same ethnic origin (Turkish). The BMI was calculated from height and weight measurements. A BMI < 18.5 kg/m^2^ was considered underweight, 18.5 to 24.99 kg/m^2^ ideal weight, 25 to 29.99 kg/m^2^ overweight, and > 30.0 obese (WHO, 2004).

The Scale for the Assessment of Negative Symptoms (SANS) and the Scale for the Assessment of Positive Symptoms (SAPS) were applied to all patients to assess the disease symptoms, and Wisconsin Card Sorting Test: Computer Version 4 (WCST: CV4) was applied for the cognitive assessment.

SANS is used to obtain a clinical rating for the negative symptoms, and SAPS is used for the positive symptoms in schizophrenia patients. SANS is a 25-item scale consisting of 5 subscales as affective blunting, alogia, apathy, anhedonia, and attention. SAPS consists of 34 items and four subscales: hallucinations, delusions, odd behavior, and positive formal thought disorder. Both scales were developed by Andreasen, and the Turkish versions’ validity and reliability were studied by Erkoc et al. [32–35].

Wisconsin Card Sorting Test is sensitive to frontal lobe functions and is used to evaluate executive functions. Cards containing different shapes and colors included in the test are given to the subject; he/she is expected to match according to color, shape, and quantity categories, but this is not told to him/her. The subject is only informed that his/her choice was ‘true’ or ‘false’. The subject is expected to understand the rules according to ‘true’ and ‘false’ feedback. WCST includes ten variables: Number of Correct Responses, Number of False Responses, Number of Completed Categories, Perseverative Responses, Perseverative Errors, Non-perseverative Responses, Trials to Complete First Category, Conceptual Level Responses, Failure to Maintain Set, and Learning to Learn. These ten variables were evaluated separately. The computer version of the test was used in this study [36].

SANS and SAPS scales and WCST: CV4 were repeated after the replacement therapy in patients whose vitamin D reached sufficient levels with the replacement therapy.

### Blood samples and laboratory analysis

Venous blood samples (10 mL) taken from the patients were into tubes, and the plasma was separated by centrifugation for 10 min at 4000 rpm. On the same day, a total of 25OHD and parathyroid hormone levels were measured with Beckman Coulter Dxi600 through the chemiluminescence method using commercial kits. A serum 25OHD level < 20 ng/mL was accepted as vitamin D deficiency, 20 to 29.99 ng/mL as vitamin D insufficiency, and ≥ 30 ng/mL as adequate [10].

### Vitamin D supplementation

Patients with vitamin D levels < 30 ng/mL were evaluated by an endocrinology and metabolism specialist. In addition to the vitamin D level, serum calcium, phosphorus, creatinine, parathyroid hormone, and alkaline phosphatase levels were evaluated. Oral replacement treatments were initiated according to the Turkish Endocrinology and Metabolism Society Diagnostic and Treatment Manual for Osteoporosis and Metabolic Bone Diseases [10]. Accordingly, 50,000 IU oral vitamin D was given once a week for eight weeks to the patients whose vitamin D levels were < 20 ng/mL (vitamin D deficiency), and 1500 IU oral vitamin D daily was given to the patients whose vitamin D level was between 20 to 29.99 ng/mL (vitamin D insufficiency). Vitamin D level was measured eight weeks after the initiation of the treatment. Patients whose serum vitamin D level could not reach > 30 ng/mL were given additional doses until the optimal level was reached.

### Statistical analyses

For statistical analyses, the SPSS 25 package program (version 25, SPSS Inc., Chicago, USA) was used. Descriptive statistics for continuous variables were given as mean ± standard deviation, and categorical variables were given as a number (n) and percentage (%). The Independent-Samples t-test was used to examine the relationship between the variables that may affect vitamin D levels and the vitamin D level and to examine whether vitamin D levels had an initial effect on the scale scores. The Shapiro-Wilk test was used to determine whether the data conformed to the normal distribution before the comparisons to examine the effect of vitamin D replacement on scale scores. The Paired-Sample t-test was used for data conforming to the normal distribution, and the Wilcoxon Signed-Rank test was used for those that did not. The Student t-test and 2 × 2 mixed pattern analysis of variance (ANOVA) were used to determine the effects of vitamin D replacement on scale scores. A series of t-tests were conducted to examine whether the SANS and SAPS subscale scores differed. A *p*-value of < 0.05 was accepted to be statistically significant.

## Results

At the beginning of the study, the mean 25OHD levels of 52 schizophrenia patients were 17.89 ± 6.22 ng/mL (*n* = 52, min: 4.26, max: 34.00); 14.39 ± 6.53 in women (*n* = 8) and 18.53 ± 6.02 in men (*n* = 44). Only 3.8% (*n* = 2) of the patients had 25OHD levels within the normal range. Approximately 96% (*n* = 50) were below the optimal vitamin D levels.

Vitamin D deficiency was found in 65.4% (*n* = 34) of the patients with vitamin D values below the normal limit, and vitamin D insufficiency was found in 30.8% (*n* = 16). Serum calcium, phosphorus, creatinine, parathyroid hormone, and alkaline phosphatase levels were within normal limits in all patients. In our study, no side effect related to vitamin D replacement was observed.

### Descriptive statistics

After several patients were excluded for various reasons, this study was completed with 40 patients (34 males and 6 females). The patients’ mean age was 41.0 ± 10.02 (ages range: 25–60), the mean serum 25OHD level was 17.87 ± 5.54 (min: 9.12, max: 28.52). The mean duration of illness was 14.00 ± 6.53 (min: 2, max: 30) years. Thirty-four patients (85%) were using atypical antipsychotics, two (5%) were using typical antipsychotics, and four (10%) were using both (Table 1).

**Table 1:** Sociodemographic and clinical characteristics of the patients

	Number (n)	Percentage (%)
**Gender**		
Male	34	85
Female	6	15
**Education**		
Literate	1	2.5
Primary school	22	55
High school	17	42.5
**Occupation**		
Unemployed	31	77.5
Employed	2	5
Retired	7	17.5
**Marital Status**		
Single	28	70
Married	11	27.5
Divorced/widow	1	2.5
**Income**		
None	14	35
Disability pension	15	37.5
Pension	10	25
Employed	1	2.5
**Somatic illness**		
None	32	80
Yes	8	20
**25OHD level**		
< 20 ng/dL - Deficiency	26	65.4
20–29.9 ng/dL - Insufficiency	14	30.8
**Total**	40	100

#### The relationship between 25OHD levels and the variables that can affect it

No significant difference was found between the 25OHD levels (deficiency or insufficiency) and the variables such as smoking, tea-coffee consumption, skin color, daily activity level, diet, clothing style, and BMI (*p* > 0.05). A statistically significant relationship was found between the duration of sunlight exposure and the 25OHD level (*p* < 0.05) (Table 2).

**Table 2:** The distribution and relationship between 25OHD levels and the variables that can affect it

	25OHD Level		
	Deficiency	Insufficiency	
	(20–29.9 ng/dL)	(< 20 ng/dL)	***p***
**Smoking (per day)**			
Nonsmoker	8	18	> 0.05
Less than one pack/day	4	6	
More than one pack/day	2	2	
**Tea/coffee consumption**			
None	1	2	> 0.05
2 large cups/day or less	4	8	
2–5 large cups/day	6	9	
5 large cups/day or more	3	7	
**Skin color**			
Light	17	11	> 0.05
Fair-skinned	6	2	
Brunette	3	1	
**Physical activity (Daily)**			
Low (< 1 h/day)	20	4	> 0.05
Moderate (1 h/day)	5	9	
Adequate (> 1 h/day)	1	1	
**Diet (Fish consumption)**			
Less than once a month	4	5	> 0.05
Once a month	5	3	
Once every two weeks	12	2	
Once to twice a week	5	4	
**Sun exposure**			
Less than one hour/day	23	6	> 0.05
1–2 h/day	3	8	
**Clothing style**			
Hijab clothing	25	12	> 0.05
Non-hijab clothing	1	2	
**Body-mass index (BMI) kg/m**^**2**^			
18.5–24.99 - Ideal weight	5	3	> 0.05
25–29.99 - Overweight	11	8	
> 30.0 - Obese	10	3	
**Total**	26	14	

### The relationship between the 25OHD level and disease severity

The 25OHD level (deficiency or insufficiency) had no significant effect on the baseline SANS and SAPS total and subscale scores (*p* > 0.05 for all scales and subscales). When evaluated separately for male and female genders, it was seen that the 25OHD level did not have a significant effect on symptom severity (*p* > 0.05). No significant relationship was found between the 25OHD level and the duration of illness or antipsychotic use (p > 0.05).

### The effect of vitamin D replacement on SANS and SAPS scores

**Fig. 1:**
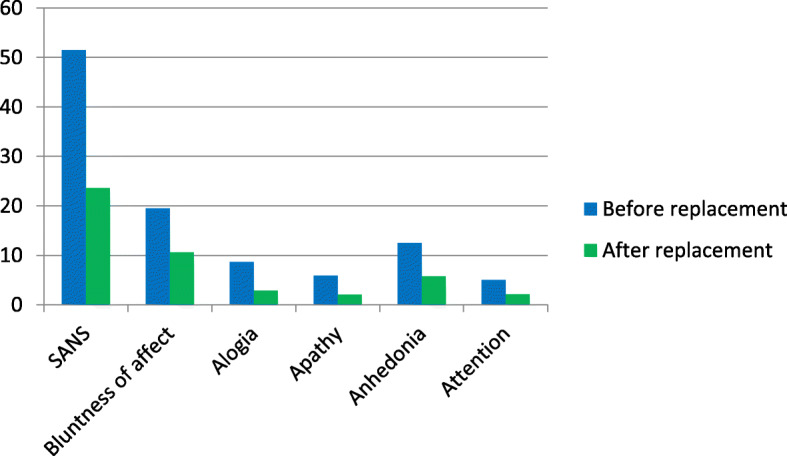
The effect of vitamin D replacement on SANS scale and subscale scores

**Fig. 2:**
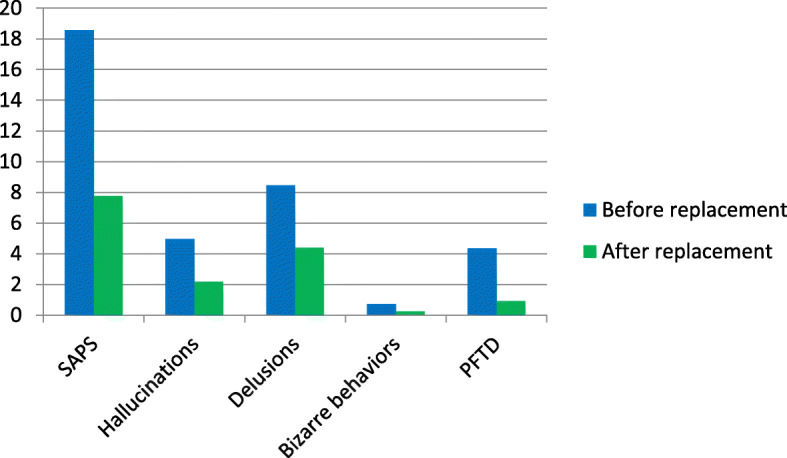
The effect of vitamin D replacement on SAPS scale and subscale scores

Participants’ mean SANS and SAPS scores after replacement (23.60 ± 15.51 and 7.78 ± 8.84, respectively) were significantly lower than those before the replacement (51.45 ± 17.96 and 18.58 ± 15.59, respectively). Statistically significant differences were found between the SANS and SAPS scale and subscale scores before and after replacement (*p* < 0.001 for SANS/SAPS total scores and all subscale scores) (Table 3, Fig. 1, and Fig. 2).

**Table 3:** The mean scale and subscale scores before and after vitamin D replacement

	Vitamin D level						
	Before replacement			After replacement			
	(< 30 ng/dL)			(> 30 ng/dL)			
	Insufficiency	Deficiency	All	Insufficiency	Deficiency	All	
	(20–29.9 ng/dL)	(< 20 ng/dL)		(20–29.9 ng/dL)	(< 20 ng/dL)		
**Scale/Subscale**	n = 16	n = 34	*n* = 40	n = 16	*n* = 34	*n* = 40	p
**SANS**	47.9 ± 17.4	53.3 ± 18.3	51.5 ± 18.0	22.8 ± 12.8	24.0 ± 17.0	23.6 ± 15.5	p < 0.001
Affective blunting	18.9 ± 6.0	19.8 ± 6.6	19.5 ± 6.3	10.6 ± 6.8	10.7 ± 6.2	10.6 ± 6.3	p < 0.001
Alogia	8.2 ± 4.8	8.9 ± 5.1	8.7 ± 5.0	2.6 ± 2.8	3.0 ± 4.2	2.9 ± 3.7	p < 0.001
Apathy	4.6 ± 3.7	6.6 ± 3.4	5.9 ± 3.6	1.6 ± 2.0	2.3 ± 3.3	2.1 ± 2.9	p < 0.001
Anhedonia	11.8 ± 4.3	12.9 ± 5.4	12.5 ± 5.0	6.0 ± 2.6	5.7 ± 4.4	5.8 ± 3.9	p < 0.001
Attention	4.6 ± 1.9	5.2 ± 2.0	5.0 ± 2.0	2.1 ± 1.7	2.0 ± 1.9	2.1 ± 1.8	p < 0.05
**SAPS**	20.0 ± 16.5	17.8 ± 20.0	18.6 ± 15.6	8.4 ± 9.4	7.5 ± 8.9	7.8 ± 8.9	p < 0.001
Hallucinations	6.0 ± 5.3	5.2 ± 6.0	5.0 ± 5.7	2.3 ± 2.7	2.1 ± 3.6	2.2 ± 3.3	p < 0.001
Delusions	7.3 ± 9.3	7.5 ± 7.3	8.5 ± 8.0	4.5 ± 5.8	4.3 ± 6.7	4.4 ± 6.3	p < 0.001
Bizarre behavior PFTD	1.5 ± 1.1 4.5 ± 8.0	0.7 ± 1.5 4.3 ± 6.2	0.7 ± 1.4 4.4 ± 6.8	0.3 ± 0.7 1.3 ± 3.8	0.2 ± 0.66 0.7 ± 3.5	0.3 ± 0.7 0.9 ± 3.6	p < 0.05 *p* < 0.01

### Comparison of WCST scores before and after the replacement therapy

The ten variables included in the WCST were evaluated separately. Learning to Learn score could not be measured in 65% of the participants before vitamin D replacement and 70% after vitamin D replacement; therefore, this variable could not be analyzed. After vitamin D replacement, no significant change was detected in the other nine variables: Number of Correct Responses, Number of False Responses, Number of Completed Categories, Perseverative Responses, Perseverative Errors, Non-perseverative Responses, Trials to Complete First Category, Conceptual Level Responses, and Failure to Maintain Set (*p* > 0.05).

Total Attention Scores (TASs), consisting of the sum of digit span test and n-back test, were calculated. The mean TAS was significantly higher after vitamin D replacement (8.60 ± 1.57) than before the replacement (8.17 ± 1.52). The effect of vitamin D replacement on TAS was significant (*p* < 0.05). No significant difference was detected between the vitamin D deficiency and insufficiency groups in terms of mean TAS after replacement (*p* > 0.05).

## Discussion

Our study is one of the few studies in the literature examining the effects of vitamin D replacement on schizophrenia symptoms. Of three other studies, one has not yet been completed [37–39]. In our study, significant improvements in total SANS, SAPS, and their subscale scores and TASs were found after vitamin D replacement compared to the values before replacement in schizophrenia patients. To the best of our knowledge, this study is the first to show improvement in negative and positive symptoms in schizophrenia patients with the treatment of vitamin D deficiency.

Previous studies in schizophrenia patients found vitamin D deficiency rates varying between 22 and 65% and insufficiency rates between 30 and 66% during the acute exacerbation period and during hospitalization [7, 19, 30, 40]. These rates were even higher in the treatment-resistant patient group (79–90%) [41]. These data have also been confirmed by meta-analysis and review studies examining vitamin D levels in patients with schizophrenia [2, 42]. In our study, 65.4% of the patients had vitamin D deficiency (< 20 ng/dL), 30.8% had vitamin D insufficiency (20–29.9 ng/dL), and only 3.8% of the patients had an optimal level of vitamin D. Similar to our study, Bulut et al. (2016) conducted a study with schizophrenia patients in an outpatient clinic. They found that vitamin D levels were deficient in 20%, insufficient in 13.75%, and sufficient in 61.25% of the patients [43]. Yuksel et al. (2014) found vitamin D deficiency in 22%, vitamin D insufficiency in 68.3%, and adequate vitamin D levels in only 9.8% of the patients. Although our study population consisted of outpatients and blood samples were taken between May and July when sunlight is more intense, the rates of low vitamin D levels were found to be high, similar to those in studies conducted during the acute exacerbation period [7]. Vitamin D synthesis varies according to geographic location and seasonal characteristics [5, 42]. Trabzon is located in northern Turkey (38° 30′ – 40° 30′ East and 40° 30′ – 41° 30′ North). Based on the data provided by the Governorship of Trabzon, the average number of sunny days during the year is very low [44]. The results obtained in this study may be due to the geographical characteristics of the city where the study was conducted.

In our study, vitamin D levels in schizophrenia patients were found to be associated with the duration of sunlight exposure. No relationship was found between vitamin D levels and other variables such as smoking, tea and coffee consumption, skin color, physical activity level, diet (fish consumption), clothing style, and BMI. The duration of sunlight exposure positively affects vitamin D levels in patients with schizophrenia [42]. A meta-analysis study found that vitamin D levels in psychotic patients were only related to the season in which blood samples were taken and parathyroid hormone levels [4]. Since 90–95% of vitamin D is synthesized in the skin through exposure to direct sunlight, other variables may not affect vitamin D levels. Our findings suggest that sunlight is more critical than other factors for vitamin D synthesis.

The trials about the association between vitamin D deficiency and disease severity in schizophrenia patients were generally conducted with patients in the acute exacerbation period, and the results vary. Although vitamin D levels were found to be low in psychotic patients, this was reported to be independent of the severity of the disease [19, 30, 45, 46]. Several studies are reporting that low vitamin D is associated with negative symptoms of schizophrenia [9, 11, 20, 47–50]. In some studies, low vitamin D levels were found to be associated with both positive and negative symptoms [7, 43, 51, 52]. No association was found between vitamin D levels and SANS and SAPS scale and subscale scores in our study. Studies reporting an association between vitamin D levels and symptom severity were usually conducted with patients in the acute exacerbation period. Since the patients included in our study were outpatients, the association between vitamin D levels and disease severity might have been missed.

Vitamin D deficiency has been reported to cause attention and memory deficiencies and cognitive impairment [30]. VDR and 1α-hydroxylase are expressed at high levels in brain areas important for cognitive functions, such as the hippocampus and cortex. Vitamin D also affects the production of various neurotransmitters, such as acetylcholine, dopamine, and serotonin [53]. Therefore, a relationship between 25OHD and cognitive functions is possible. Vitamin D deficiency is associated with various neurodegenerative disorders in which cognitive impairment is an important feature (i.e., schizophrenia, depression, attention deficit hyperactivity disorder, autism spectrum disorders, and Alzheimer’s disease) [15]. Low vitamin D in schizophrenia is associated with increased symptom severity, more severe negative symptoms, and more severe cognitive impairment (11; 48, 50).

Studies on the effect of vitamin D deficiency on cognitive functions are confusing. Low 25OHD levels have been shown to cause more severe cognitive impairment in the elderly and patients with schizophrenia [11, 54]. However, low vitamin D levels were not associated with cognitive impairment in two studies with large samples [54, 55]. It is unclear which cognitive functions are affected by vitamin D deficiency and whether chronic deficiency alters brain functions in the long term [56]. No relationship was found between the vitamin D level and TASs or WCST-CV sub-dimension scores, which may be related to the duration of vitamin D deficiency of the patients in our study. We found a significant improvement in the TASs measured by WCST after vitamin D replacement compared to those before replacement levels. Krivoy et al. (2014) conducted a double-blind placebo-controlled study and found that cognitive symptoms improved with vitamin D treatment in chronic schizophrenia patients using clozapine [37]. The improvement achieved in TASs in our study is consistent with the data in the literature, indicating that vitamin D deficiency may cause attention disorders through the frontal cortex.

We could find two studies investigating the effect of vitamin D replacement on disease symptoms [37, 38]. A recent double-blind, placebo-controlled, multicenter study in first-episode schizophrenia patients aims to evaluate the effects of high-dose vitamin D on early psychosis, but it has not been completed yet [39]. In a randomized controlled trial, Sheikhmoonesi et al. (2016) found that increased serum vitamin D levels did not improve negative or positive symptoms in male residual schizophrenia patients with low vitamin D who received fixed-dose antipsychotic therapy [38]. In a double-blind placebo-controlled study, Krivoy et al. (2014) found no improvement with vitamin D treatment in psychotic symptoms in schizophrenia patients treated with clozapine [37]. We found a significant improvement in both positive and negative symptoms after eliminating vitamin D deficiency in outpatient schizophrenia patients. In addition to its neurodevelopmental effects in the intrauterine period, vitamin D provides neuroprotection in the adult brain [57]. In recent years, studies provided increasing evidence that vitamin D provides neuroprotective, anti-inflammatory, and immunomodulatory effects by reducing oxidative stress in the brain. Animal studies supported the idea that vitamin D deficiency is associated with behavioral and cognitive impairments [58]. Low vitamin D levels may influence schizophrenia patients’ symptom severity through inflammatory effects [9].

In summary, our findings support that vitamin D levels may be low in schizophrenia outside the first episode or acute exacerbation period, and symptomatic recovery can be achieved independent of antipsychotic treatment after vitamin D level returns to normal.

Debate continues as to whether vitamin D deficiency is the cause or the result of schizophrenia. Vitamin D, which plays a role in the neurodevelopmental etiopathogenesis of schizophrenia, seems to affect the severity of schizophrenia in the period after the disease emerges. In clinical practice, the evaluation of vitamin D levels is usually overlooked. Vitamin D deficiency should be kept in mind, and vitamin D replacement should be considered as necessary in patients whose clinical findings cannot be improved despite regular use of adequate doses of drug therapy. In patients with schizophrenia, it is necessary to measure vitamin D levels regularly and keep vitamin D levels in the optimal range, in addition to the current antipsychotic treatment.

In addition to oral supplementation of low vitamin D levels, these patients should be encouraged to have more sunlight, which is an inexpensive resource to maintain the optimal vitamin D levels. Thus, the risk of metabolic and cardiovascular complications caused by vitamin D deficiency can be reduced in patients with schizophrenia who are already at risk due to reduced activity and antipsychotic drugs.

Our study has several limitations, including the small sample size and the lack of an evaluation of the type of antipsychotic when measuring vitamin D levels and evaluating the effects of replacement therapy on disease severity. Moreover, although patients using antiepileptics were excluded, the effects of other medications used for somatic diseases on vitamin D levels were not evaluated.

## Conclusions

To the best of our knowledge, this is the first study that investigated the effects of vitamin D replacement on disease symptoms. Our study provides evidence about psychotropic effects and the possible benefits of adding vitamin D to the antipsychotic treatment for psychotic symptoms. Schizophrenia causes high rates of disability, and correction of vitamin D deficiency may be a low-cost alternative, especially in patients whose symptoms do not improve despite adequate antipsychotic therapy. Our findings may be promising, especially for the negative symptoms that do not sufficiently improve in most patients. Since research examining the effect of vitamin D replacement on schizophrenia symptoms is scarce, our study’s contribution to the descriptive studies literature is significant. However, to prove that vitamin D provides a clinical improvement in schizophrenia, the data we have obtained should be repeated in placebo-controlled studies with larger sample sizes.

## Data Availability

The datasets used in the current study are available from the corresponding author Dr. Neriman ARAS on reasonable request.

## Abbreviations

25OHD: 25-hydroxyvitamin D
SANS: The Scale for the Assessment of Negative Symptoms
SAPS: The Scale for the Assessment of Positive Symptoms
WCST:CV4: Wisconsin Card Sorting Test: Computer Version 4

## Declarations

All methods were carried out under relevant guidelines and regulations.
